# Supplementary material for: Three-Dimensional Imaging for Multiplex Phenotypic Analysis of Pancreatic Microtumors Grown on a Minipillar Array Chip
Source: Cancers (Basel). 2020 Dec 7;12(12):3662. doi: 10.3390/cancers12123662 (PMC7762293; doi:10.3390/cancers12123662)
Supplement: Supplementary file 1 [file cancers-12-03662-s001.pdf]

cancers

MDPI

Article

Three-Dimensional Imaging for Multiplex Phenotypic Analysis of Pancreatic Microtumors Grown on a Minipillar Array Chip

Min-Suk Oh [1], Iftikhar Ali Khawar [2,3], Dong Woo Lee [4], Jong Kook Park [5] and Hyo-Jeong Kuh [1,2,6,*]

[1] Department of Biomedicine & Health Sciences, Graduate School, The Catholic University of Korea, 06591 Seoul, Korea; ohming1@catholic.ac.kr
[2] Department of Medical Life Sciences, College of Medicine, The Catholic University of Korea, 06591 Seoul, Korea; sakhawatali59@catholic.ac.kr
[3] Department of Urology, Samsung Advanced Institute of Health Science and Technology (SAIHST), Samsung Medical Center, Sungkyunkwan University, 06351 Seoul, Korea
[4] Departments of Biomedical Engineering, Konyang University, 35365 Daejeon, Korea; mems@konyang.ac.kr
[5] Department of Biomedical Science; Research Institute for Bioscience & Biotechnology, Hallym University, Chuncheon, 24252 Gangwon-do, Korea; jkp555@hallym.ac.kr
[6] Cancer Evolution Research Center, College of Medicine, The Catholic University of Korea, 06591 Seoul, Korea
* Correspondence: hkuh@catholic.ac.kr; Tel.: +82-2-2258-7511

Received: 19 November 2020; Accepted: 28 November 2020; Published: 04 December 2020

Supplementary materials

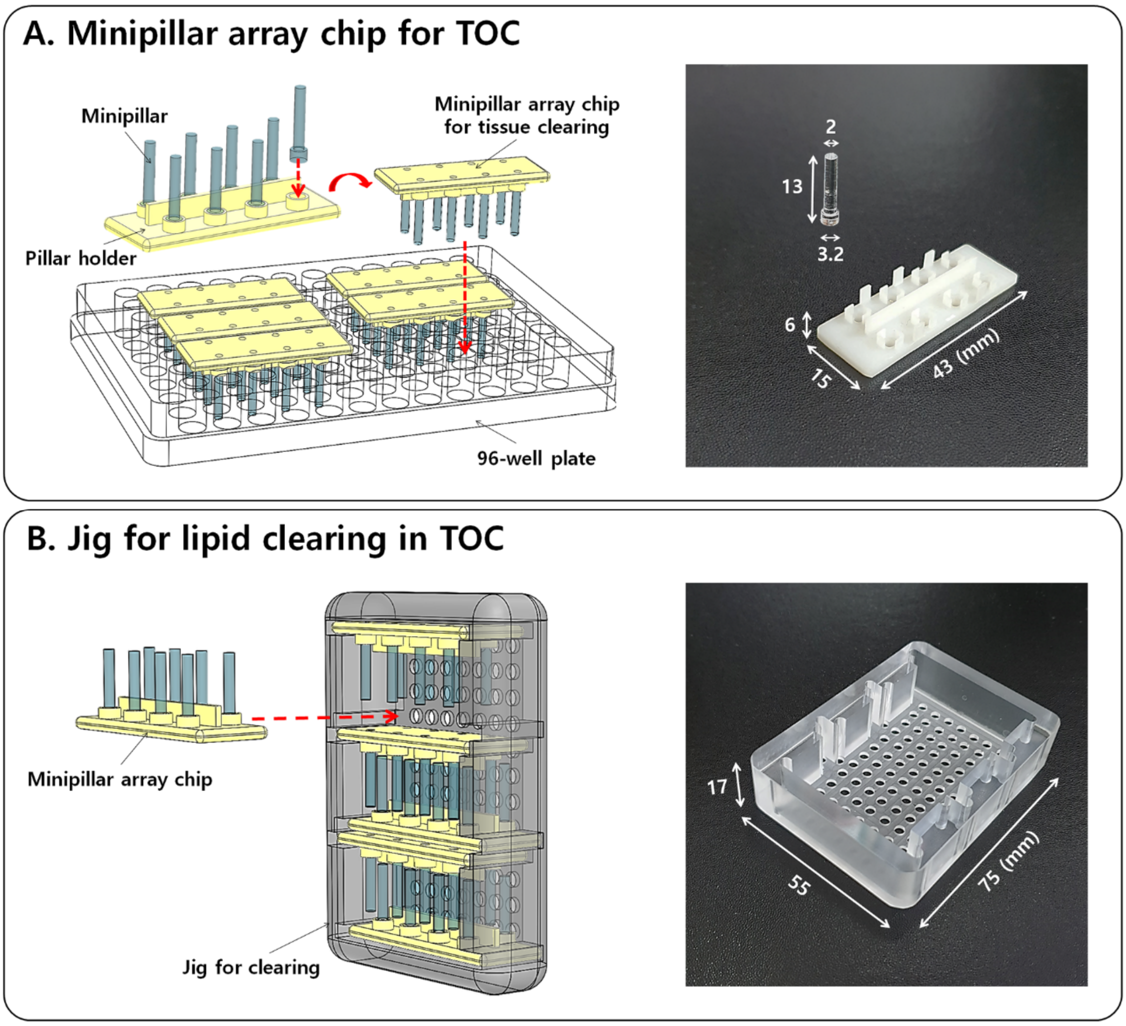

Figure S1. Schematic illustration of the minipillar array chip and jig for tissue optical clearing (TOC). (A) Maximum arrangement of six minipillar array chips (eight minipillars per chip) in a 96-well plate used during 3D culture, drug treatment, and immunostaining. (B) Assembly of six minipillar array chips in a jig for active tissue clearing using the X-CLARITY™ system.

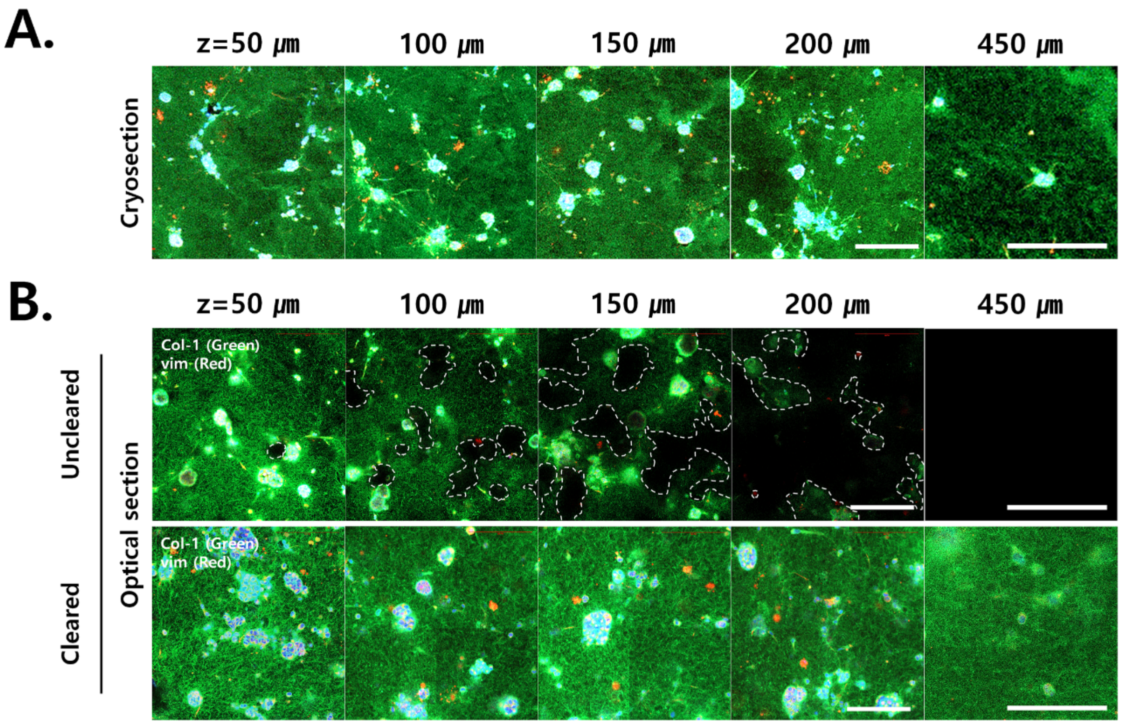

Figure S2. Comparison of fluorescence confocal images acquired from cryosections and optical sections. (A) Consecutive 10-μm-thick cryosections of microtumors (tumor spheroid-collagen construct) were prepared and images were obtained at 50-μm intervals along the z-axis. (B) Optical sections were prepared from uncleared and cleared microtumors. Optical section images were acquired at 50-μm intervals along the z-axis. Capan-1 tumor spheroids were stained with type I collagen (green), vimentin (red), and DAPI. Scale bars: 200 μm.

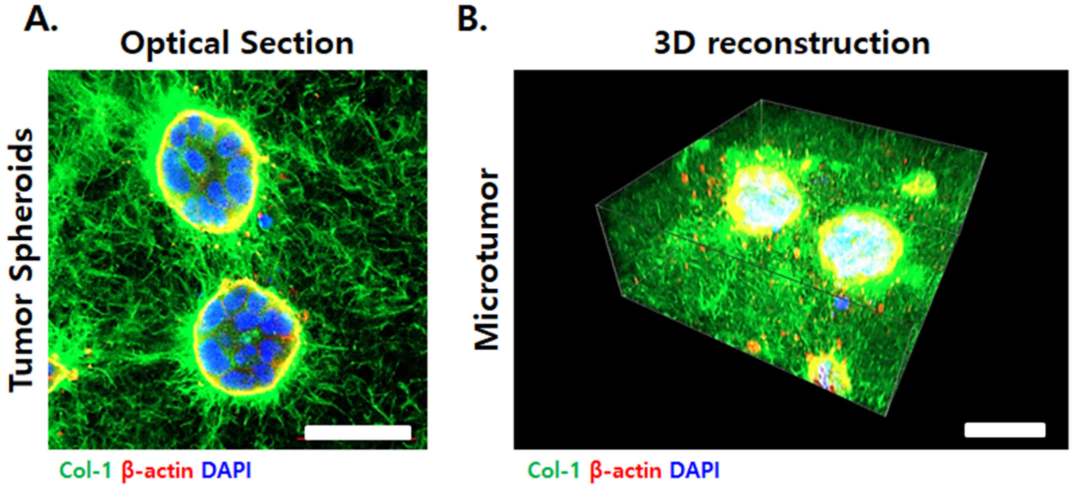

Figure S3. Fluorescence images of PANC-1 tumor spheroids (TSs) and microtumor after tissue optical clearing (TOC). (A) Fluorescence image of a Capan-1 TSs after TOC. Optical sections were obtained at z = 35 μm. (B) Three-dimensional (3D) optical images of a microtumor after TOC. Optical sections were acquired at 5-μm intervals along the z-axis for 3D reconstruction. Immunofluorescence stained with type 1 collagen (green), β-actin (red) and DAPI (blue). Scale bars: 50 μm. Col-1: type I collagen.

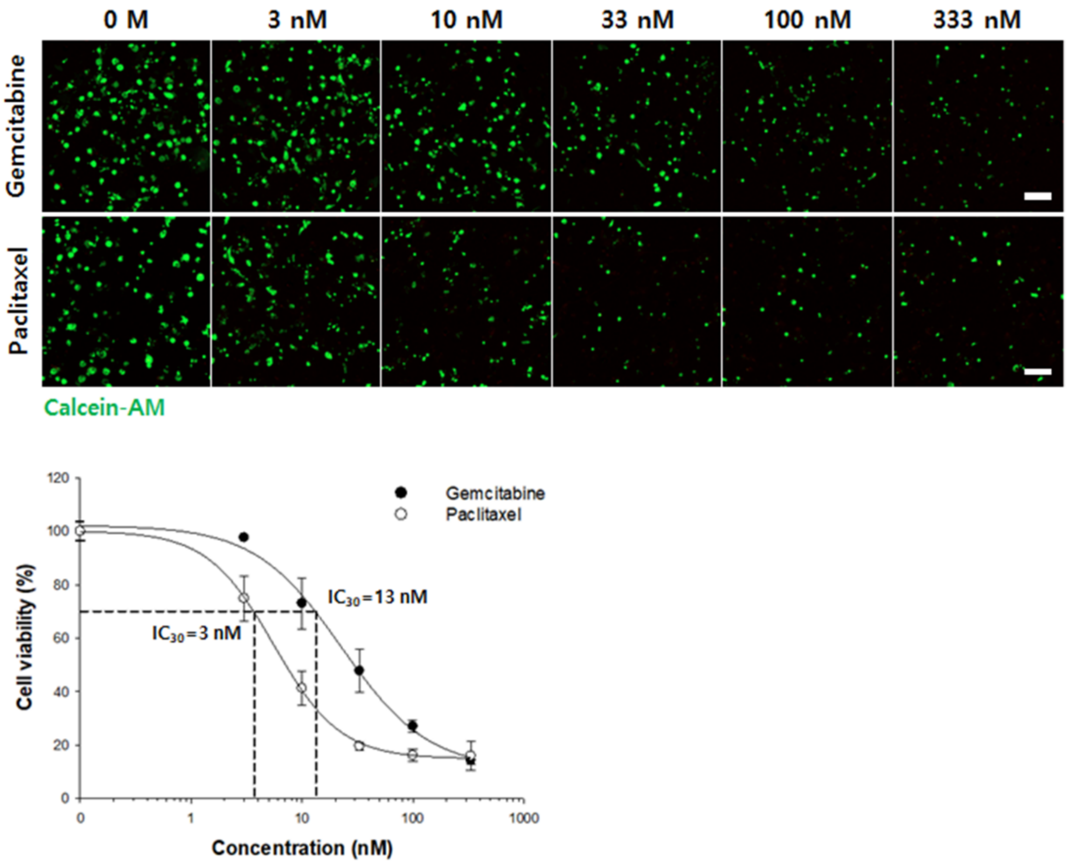

Figure S4. Dose-response curves of Gemcitabine (GEM) and Paclitaxel (PTX) in Capan-1 microtumors. Capan-1 microtumors were grown for 6 days and viability was determined using Calcein-AM staining following 48 h drug exposure. An Emax model was fitted to the data and a drug concentration showing 30% inhibition (IC_{30}) compared to the control was calculated (Sigmaplot 12.5, Systat Software, Inc., San Jose, CA). Data represent the mean ± SD of three independent experiments. Scale bars: 200 μm.

© 2020 by the authors. Licensee MDPI, Basel, Switzerland. This article is an open access article distributed under the terms and conditions of the Creative Commons Attribution (CC BY) license (http://creativecommons.org/licenses/by/4.0/).
